# Long lasting neutralization of C5 by SKY59, a novel recycling antibody, is a potential therapy for complement-mediated diseases

**DOI:** 10.1038/s41598-017-01087-7

**Published:** 2017-04-24

**Authors:** Taku Fukuzawa, Zenjiro Sampei, Kenta Haraya, Yoshinao Ruike, Meiri Shida-Kawazoe, Yuichiro Shimizu, Siok Wan Gan, Machiko Irie, Yoshinori Tsuboi, Hitoshi Tai, Tetsushi Sakiyama, Akihisa Sakamoto, Shinya Ishii, Atsuhiko Maeda, Yuki Iwayanagi, Norihito Shibahara, Mitsuko Shibuya, Genki Nakamura, Takeru Nambu, Akira Hayasaka, Futa Mimoto, Yuu Okura, Yuji Hori, Kiyoshi Habu, Manabu Wada, Takaaki Miura, Tatsuhiko Tachibana, Kiyofumi Honda, Hiroyuki Tsunoda, Takehisa Kitazawa, Yoshiki Kawabe, Tomoyuki Igawa, Kunihiro Hattori, Junichi Nezu

**Affiliations:** 1 Chugai Pharmabody Research Pte. Ltd., 3 Biopolis Drive, #07-11 to 16, Synapse, 138623 Singapore; 2 Research Division, Chugai Pharmaceutical Co., Ltd., Gotemba, Shizuoka Japan; 3 Research Division, Chugai Pharmaceutical Co., Ltd., Kamakura, Kanagawa Japan; 4 Chugai Research Institute for Medical Science, Inc., Gotemba, Shizuoka Japan

## Abstract

Dysregulation of the complement system is linked to the pathogenesis of a variety of hematological disorders. Eculizumab, an anti-complement C5 monoclonal antibody, is the current standard of care for paroxysmal nocturnal hemoglobinuria (PNH) and atypical hemolytic uremic syndrome (aHUS). However, because of high levels of C5 in plasma, eculizumab has to be administered biweekly by intravenous infusion. By applying recycling technology through pH-dependent binding to C5, we generated a novel humanized antibody against C5, SKY59, which has long-lasting neutralization of C5. In cynomolgus monkeys, SKY59 suppressed C5 function and complement activity for a significantly longer duration compared to a conventional antibody. Furthermore, epitope mapping by X-ray crystal structure analysis showed that a histidine cluster located on C5 is crucial for the pH-dependent interaction with SKY59. This indicates that the recycling effect of SKY59 is driven by a novel mechanism of interaction with its antigen and is distinct from other known pH-dependent antibodies. Finally, SKY59 showed neutralizing effect on C5 variant p.Arg885His, while eculizumab does not inhibit complement activity in patients carrying this mutation. Collectively, these results suggest that SKY59 is a promising new anti-C5 agent for patients with PNH and other complement-mediated disorders.

## Introduction

The complement system plays a crucial role in the innate immune response^1, 2^. It is widely accepted that the complement system can be activated through three distinct pathways: the classical pathway (CP), lectin pathway (LP), and alternative pathway (AP). The activation of all pathways eventually converges on the formation of the C5 convertase, which cleaves C5 to its effector subunits, C5a and C5b. C5a is an anaphylatoxin that potently induces vasodilation by stimulating histamine release from basophils and mast cells. In contrast, C5b associates with complement proteins C6, C7, C8 and C9 to assemble into a pore-forming membrane attack complex (MAC) on the surface of the target cell. Accumulation of sufficient numbers of MACs on the target cell membrane ultimately leads to colloid osmotic lysis^3^.

While a properly functioning complement system provides a robust defense against infectious microbes, inappropriate regulation of the complement system has been implicated in the pathogenesis of a variety of disorders including, e.g., lupus nephritis^4^; age-related macular degeneration (AMD)^5^; acute antibody-mediated rejection (AMR)^6^; C3 glomerulonephritis^7^; atypical hemolytic uremic syndrome (aHUS)^8^; and paroxysmal nocturnal hemoglobinuria (PNH)^9^. PNH is an acquired hematopoietic stem cell disorder characterized by mutation in the *PIGA* (phosphatidylinositol glycan class A) gene. Consequently, PNH blood cells lack glycosylphosphatidylinositol (GPI)-anchored proteins, including the complement-regulatory proteins: CD55 and CD59. The absence of those proteins causes AP activation on RBC, resulting in intravascular hemolysis by MAC. The intravascular hemolysis is associated with severe symptoms, such as anemia, dysphasia, fatigue, erectile dysfunction, thrombosis, and recurrent abdominal pain^9^. Thus, blockade of the complement cascade can provide clinical benefits to patients with PNH.

Eculizumab is a humanized monoclonal antibody against C5, and the first-in-class terminal complement inhibitor approved for the treatment of PNH and aHUS^10, 11^. Eculizumab interrupts the formation of MAC thereby compensating for the lack of CD59 on PNH erythrocytes and preventing their intravascular lysis. This results in stabilization of hemoglobin levels, reduced risk of thrombosis, and improvement in hemolysis-related symptoms, fatigue, and quality of life in patients with PNH. Although eculizumab has brought about therapeutic benefit for PNH and aHUS patients, there are several limitations to the current therapy. First, plasma C5 concentrations (80 µg/mL)^1^ are extremely high and are one of the highest amongst soluble ligand targeted by therapeutic antibody drugs^12, 13^. Treatment with eculizumab therefore requires very large doses (900–1200 mg/body) and biweekly intravenous infusion. Secondly, some patients have reported intravascular hemolysis after treatment with eculizumab, requiring the dosing interval to be further shortened to less than 2 weeks, or increased drug dosing at each biweekly interval^14, 15^. Finally, eculizumab treatment has been reported to be ineffective in patients carrying the polymorphism p.Arg885His in C5^16^.

Recently, the recycling antibody technology was developed to prolong plasma half-life of antibody and reduce the amount of drug required for therapy^17, 18^. This was achieved by introducing histidine residues into complementarity-determining regions (CDRs) or variable region of the antibody to confer pH-dependent binding to the antigen. Within the acidic milieu of the endosome, histidine residues in the antibody become protonated by the acidic pH, destabilizing antibody-antigen interactions, resulting in dissociation of the immune complex. The unbound antibodies are then recycled back to the plasma via the neonatal Fc receptor (FcRn)^19^, leaving the unbound antigen to be degraded in the lysosome. As the recycled antibody is free to bind to antigen again, this enables the lowering of antibody drug dosage and a prolongation of plasma half-life. By applying this recycling technology, we generated an anti-C5 recycling antibody, SKY59, which has significantly longer acting neutralization of plasma C5 than a conventional antibody. Using crystal structure analysis, we report that pH-dependent interaction between SKY59 and C5 is conferred by histidine residues on C5 as well as those on SKY59. This represents a novel mechanism of pH-dependent binding and serves as an alternative approach for the development of therapeutic antibodies with recycling effect. Finally, SKY59 inhibited the activity of a C5 variant (p.Arg885His), which is found in patients who respond to eculizumab poorly, representing a potential therapy for this patient subpopulation. These findings indicate that SKY59 is a promising anti-C5 agent, and is beneficial to patients with PNH and complement-mediated diseases.

## Results

### Anti-C5 antibody generation and engineering

**Figure 1:**
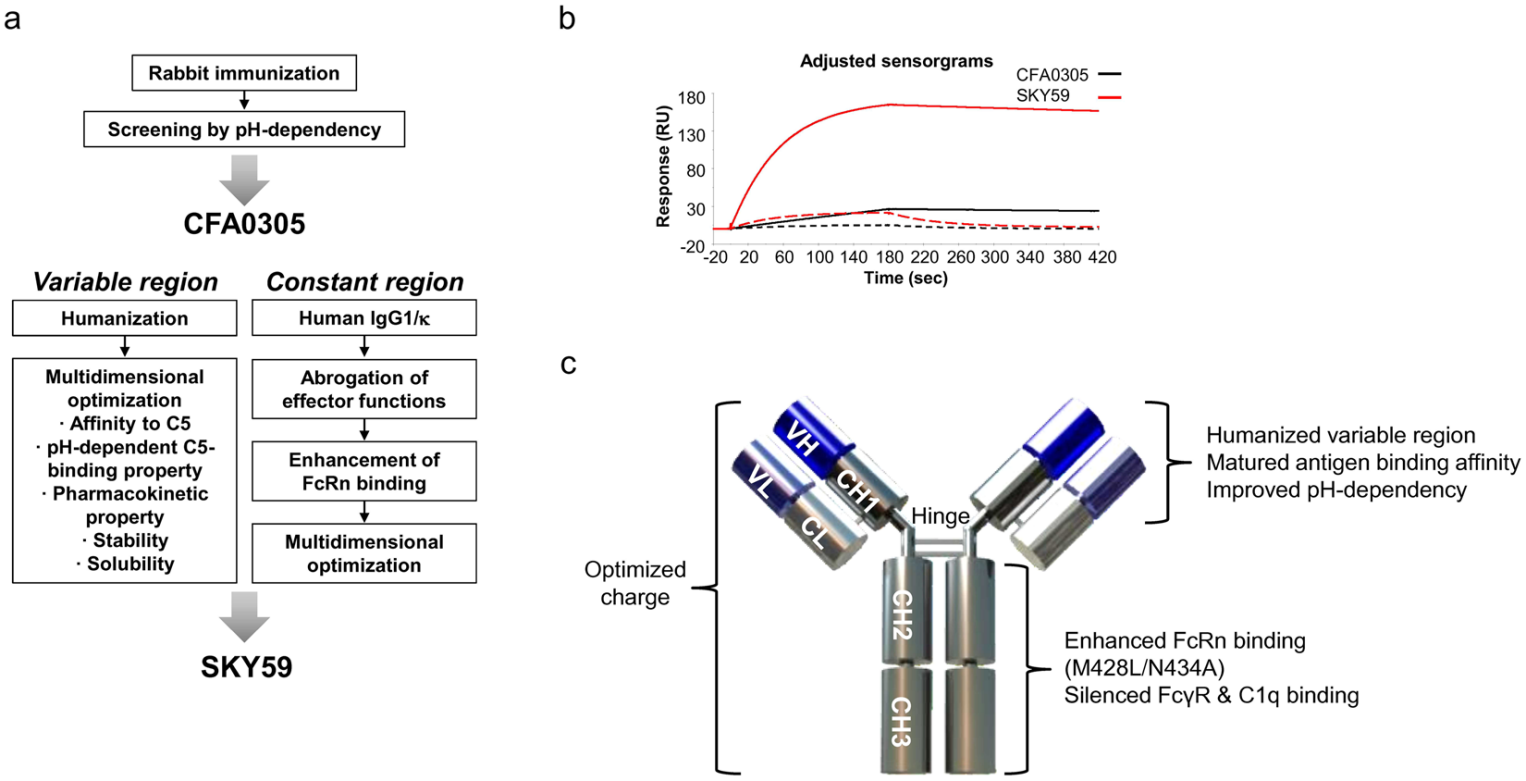
Generation of SKY59. (**a**) Antibody generation and engineering flow. A lead antibody CFA0305 with pH-dependent antigen-binding capability was obtained by rabbit B-cell cloning method. SKY59 was generated by humanization and multidimensional optimization of CFA0305. (**b**) Biacore sensorgrams of human C5 at 12.5 nM concentration binding to CFA0305 and SKY59 at pH 7.4 (solid line) and pH 5.8 (dashed line). (**c**) Schematic diagram of SKY59. Multiple mutations were applied to the variable regions and constant region of the antibody to reduce immunogenicity and to improve antigen-binding affinity, pH-dependent binding property, PK, and physicochemical properties.

The antibody generation and engineering flow is summarized in Fig. 1a. Briefly, a series of rabbit anti-C5 antibodies were generated by immunizing rabbits with human C5 protein. The screening process was designed to identify antibodies with pH-dependent antigen-binding capabilities. To accomplish this, we screened for antibodies with different binding affinities at pH 7.4 compared to pH 5.8 using ELISA and Biacore analysis. As a result, several antibodies which preferentially bind to C5 at pH7.4, but weakly at pH 5.8 were obtained. Among them, CFA0305 was chosen as a lead antibody to be further optimized based on its pH-dependent C5-binding property. After humanization of CFA0305, comprehensive mutagenesis on the antibody variable region was performed to identify mutations that improve the antigen-binding property, and combination of effective mutations gave the antibody high affinity with pH-dependent binding property (Fig. 1b). Some of histidine residues present in SKY59 were found to be important for the pH-dependent interaction.Various mutations were further applied to the variable regions of the antibody to improve pharmacokinetics (PK) and physicochemical properties, and to minimize the likelihood of immunogenicity in human^20^. The constant region of the antibody was converted to a modified human IgG1/κ without effector functions, and engineered (M428L/N434A) to have enhanced affinity to FcRn at an acidic pH to extend the plasma half-life of the antibody. This final humanized and optimized antibody was named SKY59. A summary of the multiple parameters that were engineered and optimized are shown in Fig. 1c and the details will be reported elsewhere.

### Kinetic analysis of SKY59

The affinity matured variant of CFA0305, which is SKY59, showed great improvement in binding affinity to C5 compared to CFA0305 (Table 1). Biacore analysis showed that SKY59 binds to human C5 at pH 7.4 with *K*_D_ of 1.52 × 10^−10^ M, whereas its affinity was 1,105-fold weaker at pH 5.8, *K*_D_: 1.68 × 10^−7^ M, indicating that SKY59 binds to C5 in a pH-dependent manner (Table 1). SKY59 binds human FcRn with greater affinity (*K*_D_: 1.70 × 10^−7^ M at pH 6.0) compared to trastuzumab, a control with native human IgG1 constant region (*K*_D_: 1.73 × 10^−6^ M at pH 6.0) (Table 1). Both SKY59 and trastuzumab bind FcRn very weakly at pH 7.4, which is of too low affinity to determine an accurate affinity (data not shown). There is no significant difference between human and cynomolgus monkey for binding of SKY59 to C5 and FcRn (Table 1).

**Table 1:** Binding affinity of antibodies against C5 and FcRn.

Human C5						
Antibody	pH 7.4			pH 5.8		
	*k*a (M^−1^ s^−1^)	*k*d (s^−1^)	*K*_D_ (M)	*k*a (M^−1^ s^−1^)	*k*d (s^−1^)	*K*_D_ (M)
CFA0305	9.52 × 10^4^	4.34 × 10^−4^	4.56 × 10^−9^	ND	ND	ND
SKY59	1.44 × 10^6^	2.18 × 10^−4^	1.52 × 10^−10^	8.61 × 10^4^	1.45 × 10^−2^	1.68 × 10^−7^
Cynomolgus monkey C5						
Antibody	pH 7.4			pH 5.8		
	*k*a (M^−1^ s^−1^)	*k*d (s^−1^)	*K*_D_ (M)	*k*a (M^−1^ s^−1^)	*k*d (s^−1^)	*K*_D_ (M)
CFA0305	1.05 × 10^5^	5.46 × 10^−4^	5.18 × 10^−9^	2.92 × 10^3^	1.41 × 10^−2^	4.83 × 10^−6^
SKY59	1.72 × 10^6^	3.38 × 10^−4^	1.97 × 10^−10^	1.87 × 10^4^	1.77 × 10^−2^	9.45 × 10^−7^
Human FcRn			Cynomolgus monkey FcRn			
Antibody	pH 6.0		Antibody	pH 6.0		
	*K*_D_ (M)			*K*_D_ (M)		
SKY59	1.70 × 10^−7^		SKY59	1.78 × 10^−7^		
Trastuzumab	1.73 × 10^−6^		Trastuzumab	1.98 × 10^−6^		

Binding kinetics were analyzed against C5 and FcRn using Biacore T200 instrument (GE healthcare). ND: not detected.

### C5 neutralizing activity of SKY59

**Figure 2:**
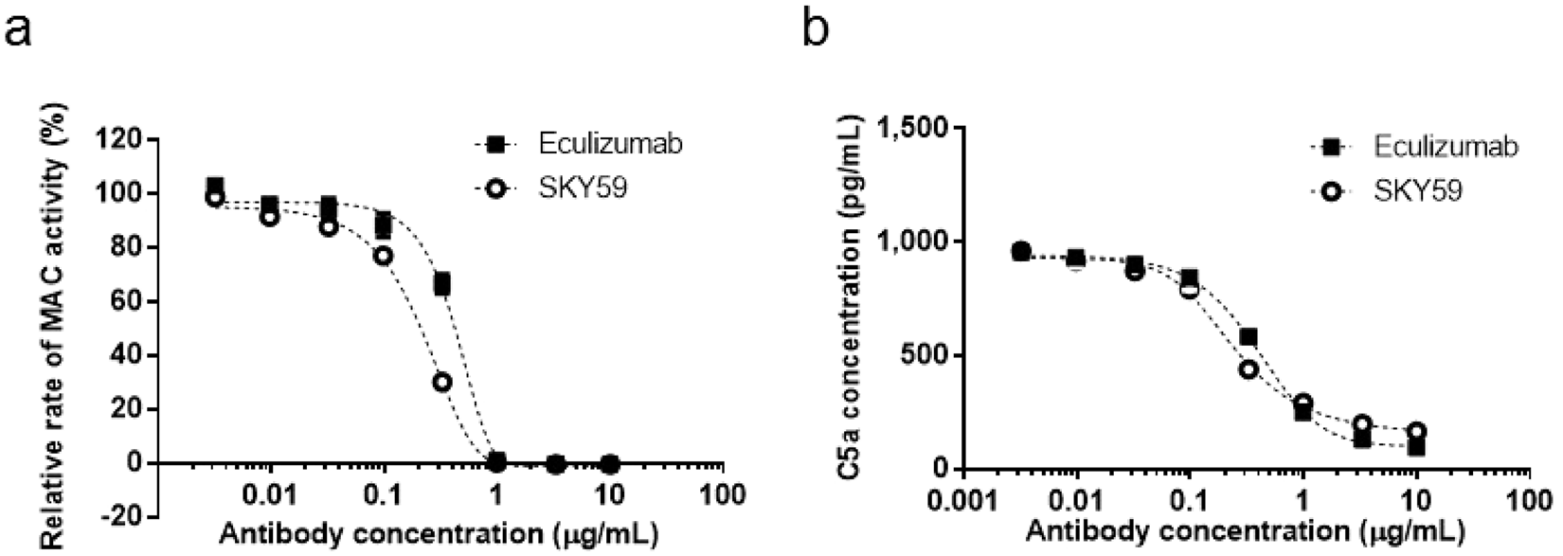
Inhibition of complement activation by eculizumab and SKY59. (**a**,**b**) Inhibitory activity of eculizumab and SKY59 on MAC formation (**a**) and C5a generation (**b**) was evaluated by a liposome-lysis assay and C5a ELISA assay, respectively. SKY59 exhibited comparable inhibitory activity to eculizumab on MAC formation and C5a generation. Data are presented as means ± S.D. The experiments were repeated twice, and representative data are shown.

SKY59 and eculizumab were tested for inhibition of complement activity *in vitro*. Liposomes sensitized with antibodies against dinitrophenyl were incubated with human serum containing the anti-C5 antibodies. Both eculizumab and SKY59 significantly inhibited the MAC-induced liposome lysis (Fig. 2a). These antibodies also inhibited C5a generation during the reaction (Fig. 2b), suggesting that SKY59 can block cleavage of C5 by the C5 convertase, with comparable inhibitory effect to eculizumab. We next examined if SKY59 could inhibit all three activation pathways in the complement system: CP, AP, and LP. As the activation of all three pathways eventually converges on the formation of C5 convertase, leading to formation of MAC, we measured C5b-9 levels after activation of each complement pathway by the Wieslab^®^ Complement assay. SKY59 inhibited C5b-9 formation significantly in all three complement pathways (Supplementary Fig. 1). EC50 values of eculizumab and SKY59 for the classical pathway were lower compared to the alternative and the lectin pathway. This would be due to the difference in serum concentration used in each assay: 0.5%, 5.5%, and 1% for CP, AP, and LP, respectively. It is therefore not possible to conclude from these assays that eculizumab and SKY59 has stronger inhibitory activity on the classical pathway than on the other pathways.

### Recycling effect of SKY59 in mice

**Figure 3:**
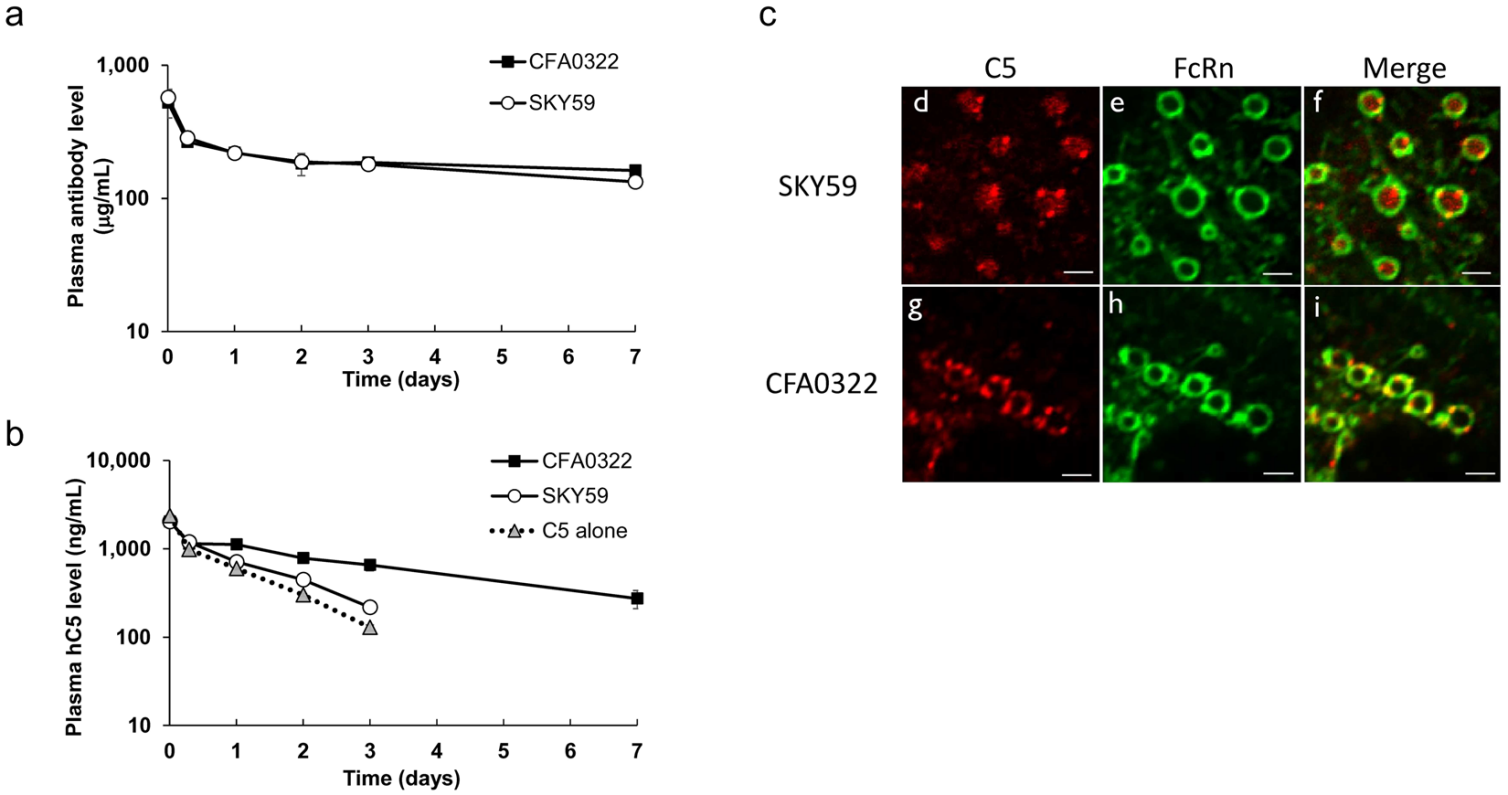
*In vivo* kinetics of C5 and anti-C5 antibody in mice. (**a**,**b**) Plasma concentrations of anti-human C5 antibody (**a**) and human C5 (**b**) were measured after intravenous administration of human C5 alone or human C5 and an anti-human C5 antibody in human FcRn transgenic mice. Data are presented as mean ± S.D. *n* = 3. (**c**) Representative images of human C5 localization in endosomal vesicles. MDCK cells expressing human FcRn-EGFP were treated with Alexa 555-labeled C5 and either SKY59 (**d–f**) or CFA0322 (**g–i**). Red and green represent C5 and FcRn, respectively. C5 was released into the endosomal space in cells treated with SKY59 while C5 remained bound on the endosomal membrane in cells treated with CFA0322. Scale bars represent 2 μm. The experiments were repeated twice for representative images in (**c**).

The *in vivo* kinetics of C5, SKY59, and CFA0322 were assessed in transgenic mice expressing human FcRn and cynomolgus monkeys. As eculizumab does not cross-react with cynomolgus monkey C5, we used CFA0322 which has an engineered human IgG4 constant region with no mutation that modifies FcRn binding. CFA0322 cross-reacts with cynomolgus monkey C5 and has a highly similar profile to eculizumab in terms of neutralizing activity and binding epitope. Similar to eculizumab^21^, CFA0322 does not bind to C5 R885H, suggesting that binding epitope of eculizumab partially overlaps with that of CFA0322 (data not shown). CFA0322 therefore serves as a surrogate for eculizumab to enable comparison against SKY59. As CFA0322 and SKY59 poorly cross-react to mouse C5, human C5 alone (0.1 mg/kg) or a mixture containing human C5 with either pH-dependent SKY59 (20 mg/kg) or non-pH-dependent CFA0322 (20 mg/kg) was administered to the mice. At this dose, the anti-human C5 antibody was present in molar excess over human C5, and therefore virtually all human C5 can be assumed to be bound to the antibody. Both CFA0322 and SKY59 exhibited comparable PK (Fig. 3a), whereas the clearance of human C5 was accelerated with SKY59 compared to CFA0322 (Fig. 3b). This implied that the C5 bound to SKY59 had dissociated from SKY59 within the acidic milieu of endosomes, while SKY59 recycled back to plasma by FcRn. To confirm this, we used Madin-Darby canine kidney (MDCK) cells stably expressing human FcRn tagged with GFP and examined under confocal microscopy whether C5 could dissociate from SKY59 within endosomal vesicles^22^. In these cells, GFP signal was located predominantly on endosomal membrane, consistent with a report that endogenous FcRn is located on the endosomal membrane and binds to IgG for recycling^23^. In order to maximize the sensitivity of this assay, the Fc portion of CFA0322 and SKY59 was replaced with an engineered Fc having strong FcRn binding in this assay. Using Alexa Fluor® 555-conjugated C5, we observed that C5 incubated with the CFA0322 variant co-localized with FcRn-GFP signal on the endosomal vesicle membrane. In contrast, C5 was seen at the center of the endosomal vesicle when incubated with the SKY59 variant (Fig. 3c). These findings suggest that SKY59 dissociates from C5 in the endosomal acidic conditions. One possible reason why 2 antibodies showed similar half-life in Fig. 3a is the insufficient study duration (7 days) to detect the difference of PK between CFA322 and SKY59. Another possible reason is the difference of Fab between CFA0322 and SKY59. PK of SKY59 without increased FcRn binding mutation might be worse than CFA0322 in mice. Therefore, it would be difficult to interpret the effect of increased FcRn binding mutation between CFA0322 and SKY59 in mice.

### Recycling effect of SKY59 in cynomolgus monkeys

**Figure 4:**
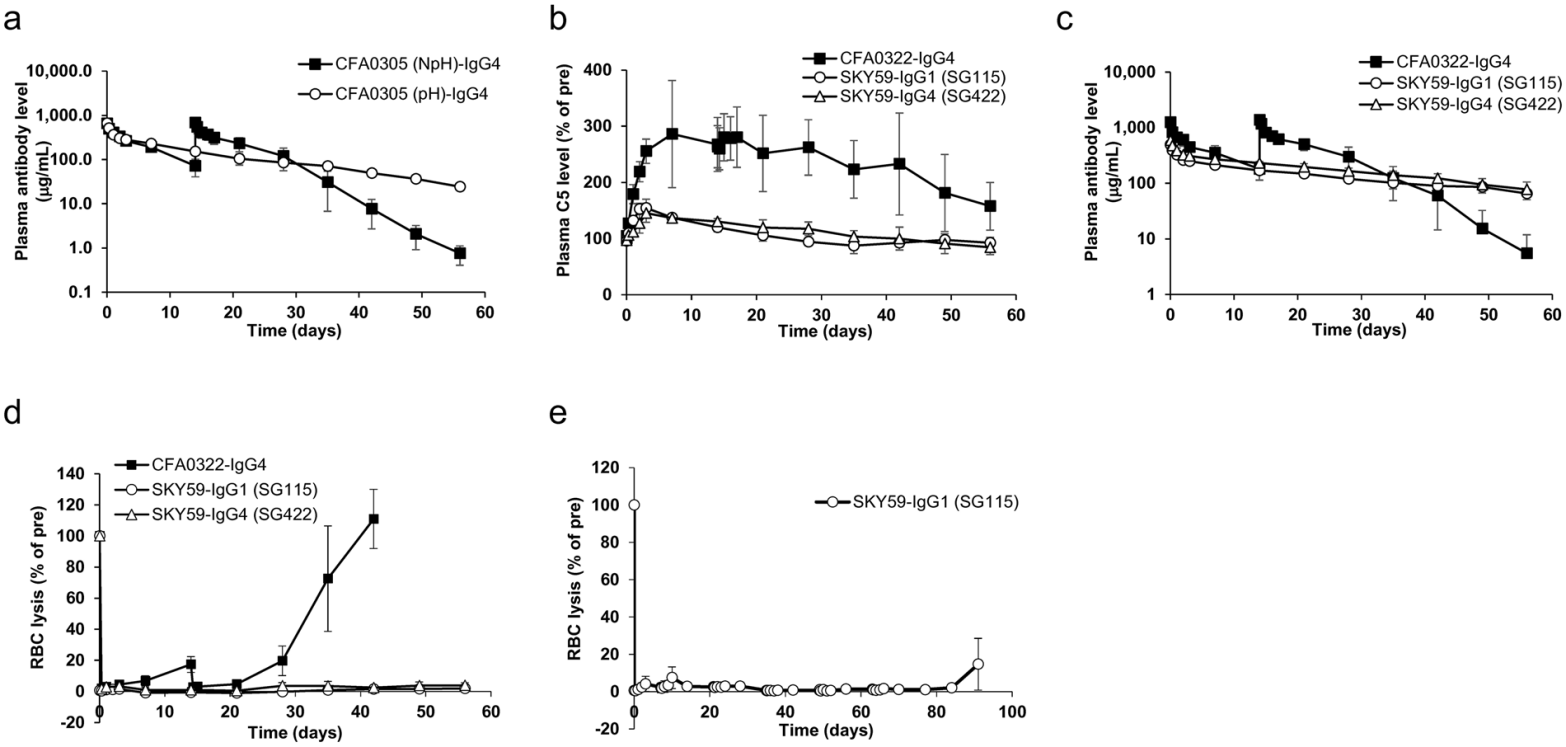
Recycling effect of anti-C5 antibody in cynomolgus monkeys. (**a**) Cynomolgus monkeys were dosed with pH-dependent C5 binding antibody (CFA0305 (pH)-IgG4) (20 mg/kg) at day 0 or non-pH-dependent antibody (CFA0305 (NpH)-IgG4) (20 mg/kg) at day 0 and 14 intravenously. Plasma samples were collected until day 56 after the first injection. Plasma anti-C5 antibody level was determined by ELISA. Data are presented as mean ± S.D. n = 2–4. (**b**–**d**) Cynomolgus monkeys were dosed with SKY59-IgG1 (SG115) (20 mg/kg), SKY59-IgG4 (SG422) (20 mg/kg) at day 0 or CFA0322-IgG4 (40 mg/kg) at day 0 and 14 intravenously. Samples were collected until day 56 after the first injection. (**e**) Cynomolgus monkeys were dosed with 5 mg/kg of SKY59-IgG1 (SG115) intravenously at day 0 and 2 mg/kg of SKY59 subcutaneously at day 7, 21, 35, 49, and 63. Plasma C5 level (**b**), anti-C5 antibody level (**c**) and complement activity (**d**,**e**) were determined after administration of anti-C5 antibody. (**b**,**d**,**e**) Data are plotted as the percentage remaining compared to the values at pre-administration. Data are presented as mean ± S.D. *n* = 2–4.

First, we evaluated the effect of pH-dependent binding to endogenous C5 on the antibody PK in cynomolgus monkeys. A non-pH dependent and pH-dependent CFA0305, which are the prototype of SKY59, were administered into cynomolgus monkeys. The constant region of CFA0305 is engineered IgG4 with no mutation that modifies FcRn binding. Figure 4a shows that PK of the pH-dependent antibody is significantly enhanced compared to the non-pH-dependent antibody. This indicates that the pH-dependent binding property contributes to the prolongation of half-life of the C5 antibody in cynomolgus monkeys. Next, we evaluated the recycling effect of SKY59 in cynomolgus monkeys. This study was designed to show that the recycling technology enables dose reduction, and that SKY59 can neutralize complement activity significantly longer even at a lower dosage than an eculizumab-like antibody, CFA0322. The dose of CFA0322 at 40 mg/kg at day 0 and 14 was chosen by referring to the expected maximum therapeutic regimen of eculizumab which is 40 mg/kg, biweekly. To demonstrate a reduction in dosing amount and frequency, SKY59 was therefore administered at a lower dose at 20 mg/kg, and only once at day 0. Since the constant region of SKY59 is a modified human IgG1 (SG115), we also tested SKY59 with human IgG4 (SG422), which has similar modifications, as the control. As commonly observed with antibodies against soluble targets^24, 25^, treatment with CFA0322 led to three-fold accumulation of plasma C5, while this accumulation was completely prevented in cynomolgus monkeys treated with SKY59 (Fig. 4b). PK of SKY59 was also significantly improved compared to CFA0322 (Fig. 4c), which is consistent with previous observation with CFA0305 (Fig. 4a). SKY59-IgG1 (SG115) showed slower clearance: 1.87 mL/day/kg, compared with CFA0322: 5.82 mL/day/kg, which is similar to the clearance of eculizumab in human: 7.54 mL/day/kg^26^. Because the affinity of SKY59 to cynomolgus monkey FcRn at acidic pH was enhanced by engineering, SKY59 was expected to be recycled by FcRn more effectively than CFA0322, which would also contribute to a longer half-life. Complement activity was monitored by measuring the plasma hemolytic activity using chicken red blood cells (cRBC). Consequently, a single intravenous administration of SKY59 stably suppressed complement activity in plasma for 8 weeks, whereas CFA0322 was no longer effective at 5 weeks despite multiple administrations at a higher dose (Fig. 4d). As the prolonged plasma half-life of SKY59 would enable dose reduction, we sought to investigate if SKY59 could be administered as subcutaneous regimen. After an initial intravenous loading of SKY59 at 5 mg/kg, SKY59 was subcutaneously administered into cynomolgus monkeys every two weeks at 2 mg/kg till day 63 (Fig. 4e). As over 20% of serum hemolytic activity in the cRBC lysis assay was reported to correlate with events such as dysphagia and dramatic increases in lactate dehydrogenase (LDH) and alanine aminotransferase (ALT) levels in PNH patients^27^, this suggests that subcutaneous administration of SKY59 in humans may effectively suppress hemolytic activity in PNH patients.

### Epitope analysis

**Figure 5:**
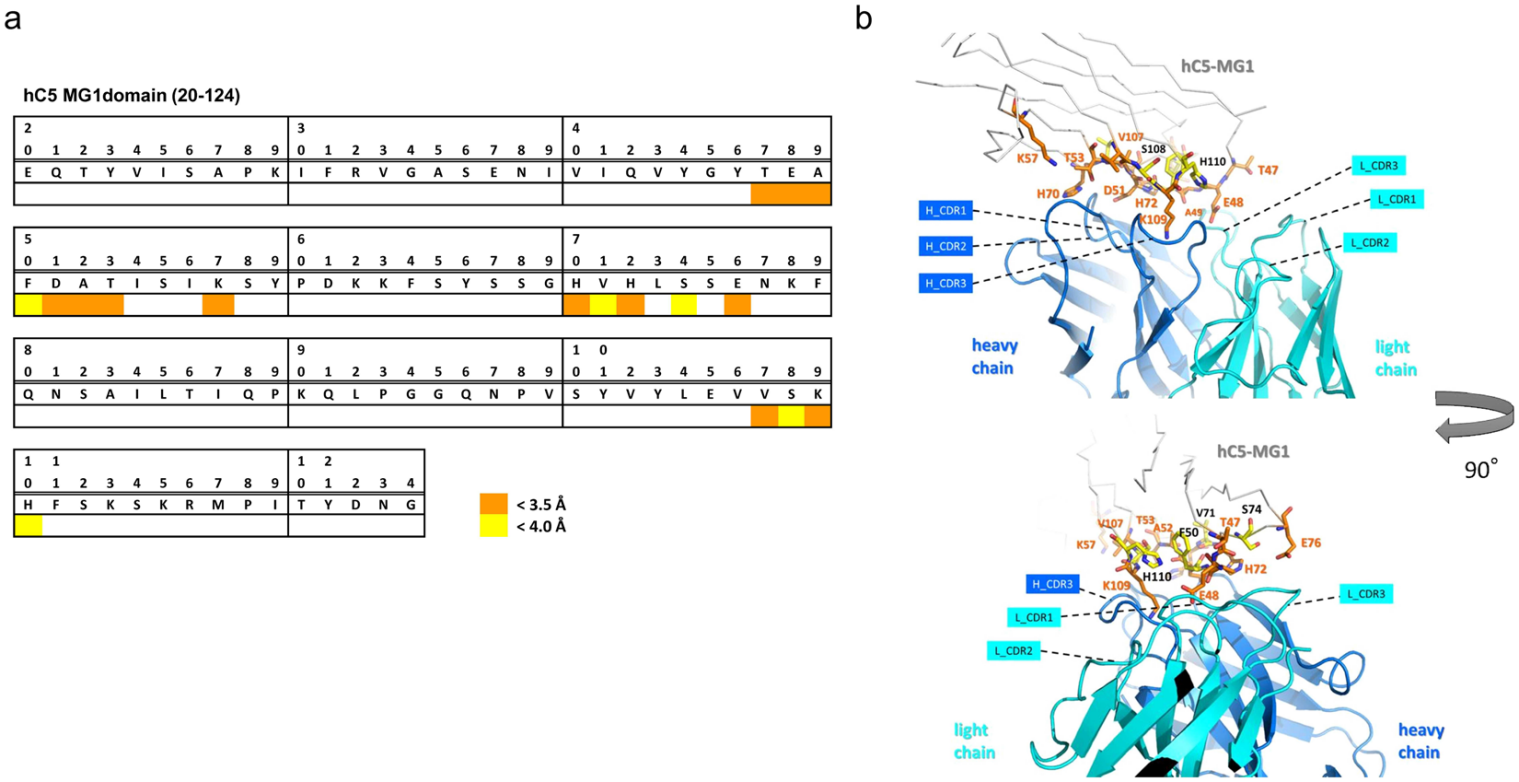
Epitope of the SKY59. (**a**,**b**) The region where C5 contacts SKY59 Fab was mapped in the MG1 amino acid sequence (**a**) and in the crystal structure (**b**). In the epitope, residues of MG1 that have at least one atom located within 4.0 and 3.5 Å from the SKY59 Fab are marked with yellow and orange, respectively. SKY59 Fab is represented by ribbons colored blue for the heavy chain and cyan for the light chain, and MG1 is represented by gray ribbons and the epitope residues shown as sticks in (**b**).

**Figure 6:**
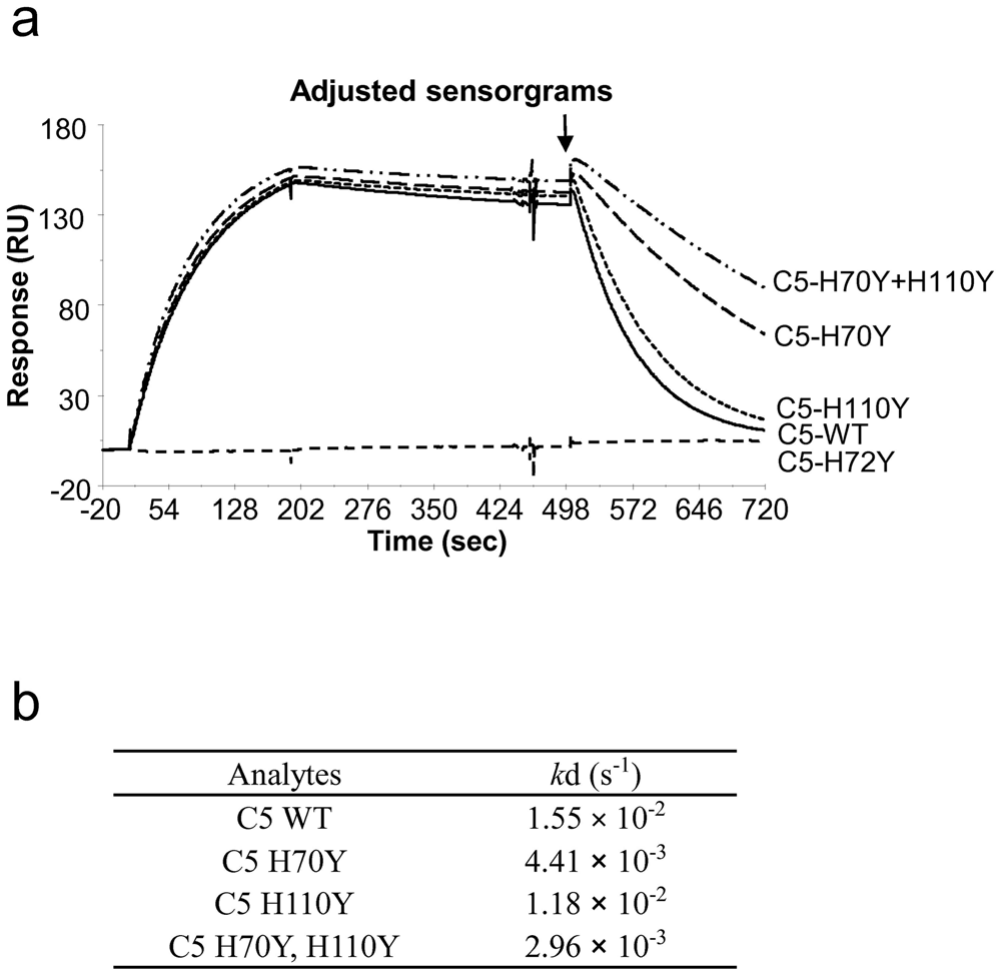
Interaction of SKY59 with C5 histidine mutants. (**a**) Biacore sensorgrams were obtained by injection of C5 WT (solid curve), C5 H70Y (long-dashed curve), C5 H72Y (short-dashed curve), C5 H110Y (dotted curve), and C5 H70Y + H110Y (dashed doted curve), respectively, over sensor surface captured with SKY59. The antibody-antigen complexes were allowed to dissociate at pH 7.4, followed by additional dissociation at pH 5.8 (pointed by an arrow) to assess the pH-dependent interactions. (**b**) The dissociation rate constant (*k*d (1/s)) of SKY59 against C5 histidine mutants at pH 5.8.

In order to clarify the mechanism of pH-dependent interaction between SKY59 and C5, we investigated the epitope of SKY59 by western blot analysis. Eculizumab bound to the α-chain of C5^28^, whereas SKY59 bound to the β-chain of C5 wild-type (WT). Further analysis using truncated mutants of C5 β-chain indicated that SKY59 bound to MG1 domain (20–124) of human C5 (data not shown). To determine the interaction mode more precisely, X-ray crystal structure analysis of the complex of SKY59 Fab fragment and the MG1 domain was performed. The diffraction intensity data up to 2.11 Å resolution were obtained, and the crystallographic data statistics are shown in Supplementary Table 1. The crystal structure of the SKY59 Fab and MG1 domain complex revealed that the three amino acids, E48, D51, and K109, of MG1 domain are located within 3.5 Å from SKY59 Fab (Fig. 5a and b)^29^. It was suggested that these three amino acids form a number of hydrogen bonds with the Fab and contribute to the antibody-antigen binding (Supplementary Fig. 2a). To confirm this, we generated human C5 point mutants, in which either E48, D51, or K109 was substituted to alanine. By Biacore analysis, all three mutations resulted in a drastic loss of affinity, demonstrating that these amino acid residues are critically involved in the antibody-antigen interaction (Supplementary Fig. 3a). Conversely, all three point mutants bound to eculizumab with a similar binding profile compared to C5 WT, confirming that the epitope of eculizumab is different from that of SKY59. These results were also confirmed by western blot analysis (Supplementary Fig. 3b). The crystal structure analysis also revealed that three histidine residues on human C5, H70, H72, and H110, were involved in the interaction with SKY59 Fab (Supplementary Fig. 2b). We hypothesized that these histidine residues were likely to be involved in the pH-dependent protein–protein interaction. To investigate this, we generated H70Y, H72Y, and H110Y human C5 point mutants and characterized their contribution to pH-dependent binding by Biacore binding analysis. The H110Y mutation resulted in a slightly slower dissociation rate while the H70Y mutation exhibited almost three-fold slower dissociation rate at pH 5.8 compared to C5 WT (Fig. 6a and b). Furthermore, a double mutation at both H70 and H110 resulted in a greater effect on pH-dependent binding with a dissociation rate at pH 5.8 almost five-fold slower than C5 WT. The H72Y mutation resulted in the complete loss of binding of SKY59 Fab to C5, suggesting that H72 is crucial for the binding to SKY59 at neutral pH.

### Neutralizing activity of SKY59 on C5 variants

**Figure 7:**
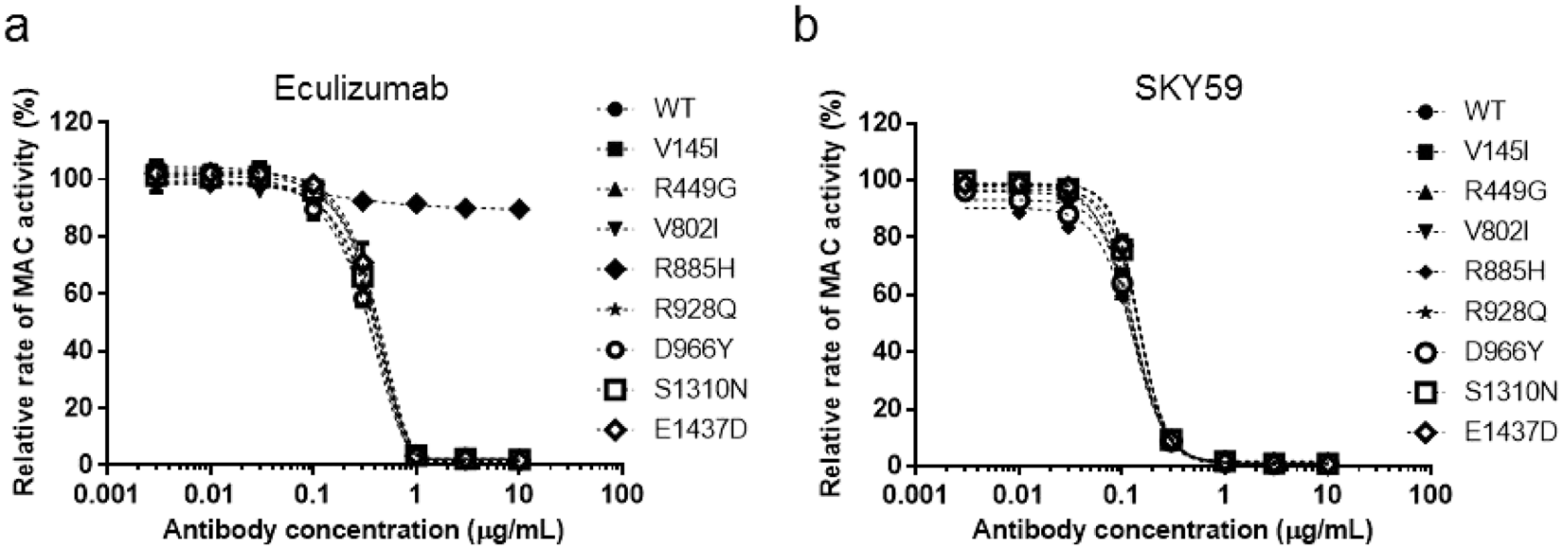
Neutralizing activity of anti-C5 antibody on C5 with non-synonymous SNPs. (**a**,**b**) Liposome lysis was caused by mixing sensitized liposome, C5-depleted serum, and either of C5 variants: V145I, R449G, V802I, R885H, R928Q, D966Y, S1310N, and E1437D. Eculizumab did not inhibit activity of R885H C5 (**a**), whereas SKY59 inhibited all C5 variants (**b**). Data are presented as means ± S.D. The experiments were repeated twice, and representative data are shown.

It is reported that a small number of PNH patients respond poorly to eculizumab. Nishimura *et al*. recently demonstrated that these patients carry a common single nucleotide polymorphism (SNP) in C5 where arginine at position 885 is substituted with histidine^16^. Since arginine at 885 in the α-chain of C5 is within the binding epitope of eculizumab^11, 21^, this C5 variant cannot be neutralized by eculizumab. In contrast, SKY59 could be expected to neutralize the activity of this C5 variant because the epitope of SKY59 is distinct from that of eculizumab. We therefore generated recombinant C5 protein with this substitution, R885H, and examined the neutralizing activity of SKY59. Consistent with previous reports, eculizumab was unable to neutralize the activity of C5 R885H, whereas SKY59 neutralized this C5 variant with a similar dose-response compared to C5 WT (Fig. 7). We further analyzed highly prevalent non-synonymous SNPs of C5. Seven kinds of C5 variants, V145I, R449G, V802I, R928Q, D966Y, S1310N, and E1437D, were selected based on their frequency using 1000 Genomes Browsers (http://www.1000genomes.org/1000-genomes-browsers). All C5 variants were shown to have comparable lytic activity to C5 WT. SKY59 exhibited neutralizing activity to all types of C5 variants tested, suggesting that the activity of SKY59 will not be affected by these highly frequent SNPs. It is also the case for eculizumab, except for C5 R885H (Fig. 7).

## Discussion

In the present study, we have generated a novel recycling antibody, SKY59, which binds to C5 in a pH-dependent manner. Due to its prolonged half-life, a single administration of SKY59 (20 mg/kg) was able to suppress complement activity in cynomolgus monkeys for up to two months. In contrast, multiple administration of a non-pH-dependent conventional antibody lost efficacy after one month, despite a two-fold higher dose. As the enhanced plasma half-life of SKY59 enabled a reduced dosing, we also demonstrated that SKY59 can suppress C5 activity when administered subcutaneously at 2 mg/kg every two weeks after an initial intravenous loading at 5 mg/kg. These data suggest that SKY59 can reduce the dosing frequency for patients with complement-related diseases, and can potentially be administered subcutaneously at biweekly intervals for greater compliance and convenience.

Before the development of anti-C5 therapeutics, the treatment of PNH was largely limited to transfusion of blood or packed RBC. With the development of eculizumab, the standard of care for PNH patients was dramatically improved, and the risk of patients developing thromboembolism was significantly reduced^30^. The drawback, however, is that eculizumab therapy requires frequent intravenous infusion at a high dose. This is unavoidable due to two factors. One is the high plasma levels of C5, which is approximately 440 nM (80 µg/mL)^31, 32^. The second is that the natural elimination rate of C5 would be reduced once it has bound to an antibody, and thus total plasma C5 levels are expected to increase due to accumulation of C5-antibody complexes. In the case of cynomolgus monkeys, we observed nearly three-fold accumulation of total plasma C5: 1320 nM. Assuming that the antibody binds to C5 in a divalent manner, at least 660 nM (99 µg/mL) of antibody would be needed to completely neutralize the three-fold accumulated C5. This level is very similar to the expected trough concentration of eculizumab of 100 µg/mL^33^. In contrast, SKY59 is able to overcome these limitations by virtue of its recycling properties. As SKY59 has pH-dependent binding to C5, it dissociates from C5 within acidic endosome vesicles and can be recycled back to the plasma by FcRn as a ‘free’ antibody to bind more C5. This property enables one SKY59 molecule to repeatedly bind and neutralize multiple C5 molecules, and therefore a much lower trough concentration is needed to completely neutralize C5 in plasma.

SKY59 differs from CFA0322 in at least three parameters that could influence PK, which are constant region of the antibody (IgG1 vs. IgG4), pH-dependent binding property to C5, and point mutations in Fc. In this study, no significant difference in PK profile was observed when SKY59-IgG1 was compared against SKY59-IgG4. On the other hand, PK of the pH-dependent antibody CFA0305 was significantly enhanced compared to the non-pH-dependent antibody, indicating that the pH-dependent binding property mainly contributes to the prolongation of half-life of the C5 antibody. Therapeutic antibodies against membrane proteins are known to exhibit nonlinear PK due to antigen internalization from the cell surface. While target-mediated antibody clearance is typically observed for drug targets expressed at the cell surface, this phenomenon is also observed for soluble targets such as PCSK9^18^. Antibody bound to soluble PCSK9 is more rapidly cleared from plasma than free antibody via the clearance mechanism inherent to PCSK9. This results in PCSK9-mediated antibody clearance. Our data suggests that this is also the case for C5. Although the detailed molecular mechanism underlying C5-mediated clearance remains unknown, after anti-C5 antibody/C5 complex is taken up into cells, anti-C5 antibody that remains bound to internalized C5 in the endoxome is expected to undergo lysosomal degradation, similar to anti-PCSK9 antibody. This results in poor PK and necessitates higher doses or more frequent administration to maintain the pharmacodynamic effect. This antigen-mediated clearance can be minimized by pH-dependent C5 binding because anti-C5 antibody/C5 complex would dissociate in the endosome, and free anti-C5 antibody would be recycled back to plasma by FcRn-mediated recycling mechanism, while free C5 would undergo lysosomal degradation. Similarly, PCSK9-mediated antibody clearance was minimized by engineering pH-dependent PCSK9 binding^18^. In addition, the mutations in Fc to enhance FcRn binding would contribute to the PK prolongation. Numerous examples have been described in which enhancement of interaction between IgG and FcRn at acidic pH leads to improved PK properties in both preclinical species^19, 34^ and humans^35^. The same Fc mutations (M428L/N434A) used in this study were also demonstrated to improve PK profile of antibody compared to intact IgG1 in preclinical studies^36^.

It is technically challenging to establish assays to detect only “free antibody” by ELISA due to shift in equilibrium of antibody-antigen complex during assays and detection of partial free antibody (one antibody arm binds to C5 and another antibody arm is free). Therefore, total antibody concentration was measured in *in vivo* studies. There is no direct evidence that SKY59 was recycled back as “free antibody”. However, since dissociation of C5 from C5-SKY59 complex in endosome was shown in *in vitro* assay in Fig. 3c, free SKY59 can be recycled back to the plasma by FcRn theoretically. In the cynomolgus monkey study, RBC lysis was evaluated using monkey plasma samples to confirm presence of pharmacologically active antibody against C5. Therefore, although antibody concentration was detected as total human IgG, the antibody should be pharmacologically active during the study.

The first therapeutic recycling antibody was created by engineering tocilizumab (Actemra)^17^, an antibody against the interleukin-6 receptor (IL-6R)^37^. Igawa *et al*. systematically substituted all residues within the CDR and framework to histidine in order to identify mutations that conferred pH-dependent binding to IL-6R. By combining multiple mutations in both the heavy and light chains, they generated an antibody with strong pH-dependent binding to IL-6R. In contrast, CFA0305, the lead antibody of SKY59 derived from rabbit immunization, was identified during the screening process to have pH-dependent binding to C5 without any prior engineering to the molecule. In order to clarify the mechanism of this pH-dependent interaction, we performed crystal structure analysis of the SKY59-MG1 complex. Interestingly, we observed that 3 histidine residues on human C5, H70, H72, and H110, are located at the antibody-antigen interface. C5 with mutations at H70Y and/or H110Y showed slower dissociation from SKY59 at pH 5.8, demonstrating that these residues are important for pH-dependent binding (Fig. 6b). From the crystal structure, we surmise that protonation of these histidine residues at low pH (i) breaks a hydrogen bond between H70 and T53 in MG1 and (ii) induces repulsion between H110 in MG1 and H-CDR3_H100c (Supplementary Fig. 2b), resulting in conformational changes that contribute to the pH-dependent antibody-antigen interaction. On the other hand, H72 is critical for the binding of C5 to SKY59. This residue of C5 is located in the pocket formed by the H-CDR2 loop of SKY59 Fab and the loop of MG1 (L73-E76). H72 fills the space in this pocket tightly and makes a hydrogen bond with H-CDR2_Y58 (Supplementary Fig. 2b). The H72Y mutation could not be tolerated as there is not enough space to accommodate the bulkier side chain of tyrosine. To our knowledge, this is the first report demonstrating that an antibody can exhibit the recycling effect *in vivo* by exploiting changes in the electrostatic charge on the antigen side. Since some of histidine residues in the variable region of SKY59 were also found to be important for the pH-dependent binding property, histidine residues present both on C5 and SKY59 variable region contribute to this pH-dependent interaction. This suggests that by combining an appropriate screening strategy and comprehensive mutagenesis on antibody variable region, antibodies with potent recycling properties can be identified against target antigens with exposed histidine residues.

Poor responders to eculizumab, defined as sustained elevated markers of hemolysis during treatment with eculizumab, have been found in a subgroup of PNH patients^16^. These patients have genetic variants which predict the polymorphism p.Arg885His. This polymorphism occurs in approximately 3.5% of Japanese and 1% of Han Chinese populations, and interferes with binding of eculizumab to C5. Recently, the first patient with no known Asian ancestry and with no response to eculizumab was found^38^. This patient carried a single C5 heterozygous missense mutation, c.2653 C > A, which predicts R885S. As the epitope of SKY59 is distant from arginine at 885, SKY59 is able to neutralize activity of C5 with SNPs at Arg885, indicating that SKY59 will be beneficial for patients carrying the genetic polymorphisms in C5 described above.

In conclusion, SKY59 is a novel recycling antibody that achieves long-lasting neutralization of C5 in preclinical models. SKY59 can provide a new therapeutic option for PNH patients in terms of reduced intravenous drug dosage, dosing frequency and even the possibility of subcutaneous administration. It is also able to provide therapeutic benefit for eculizumab resistant PNH patients carrying the R885H polymorphism and therefore represents a promising therapy for complement-mediated diseases. Human clinical trials are currently underway and could provide the necessary data to bring this novel anti-C5 agent to patient care.

## Methods

### Neutralizing activity of anti-C5 antibody

The anti-C5 antibodies were tested for inhibition of MAC activity by a liposome lysis assay. Thirty microliters of normal human serum (6.7%) (SER018, Biopredic) was mixed with 20 µL of diluted antibody in a plate and incubated for 30 min at 25 °C. Liposomes sensitized with dinitrophenyl (Autokit CH50, 995-40801, Wako) were transferred into each well. Substrate solution was added to each well. The final mixture was incubated for 40 min at 37 °C, and thereafter OD at 340 nm was measured. The relative rates (R) of lytic activity was calculated from the following formula: R (%) = 100 × [(OD_Antibody_ − OD_Serum and liposome background_)]/[(OD_Without antibody_ − OD_Serum and liposome background_)]. The C5a level in the supernatants from liposome lysis assay was quantified by using C5a ELISA kit (DY2037, R&D systems). The anti-C5 antibodies were tested for inhibition of C5 variants by a liposome lysis assay. Ten microliters of C5-deficient human serum (C1163, Sigma) was mixed with 20 μL of diluted antibody and 20 μL of cell culture medium containing recombinant C5 variant (0.02–0.03 μg/mL) in a plate and incubated for 30 min at 37 °C. Liposomes and substrate solution were transferred into each well. The final mixture was incubated for 90 min at 37 °C, and thereafter OD at 340 nm was measured.

### Biacore binding assays

Binding kinetic of anti-C5 antibodies against human C5 or cynomolgus monkey C5 were assessed at pH 7.4 and pH 5.8, at 37 °C using Biacore T200 instrument (GE Healthcare). Antibody was captured onto the Biacore C1 sensor surface that was immobilized with ProA/G. Recombinant human C5 or monkey C5 was prepared by two-fold serial dilution started from 12.5 nM for SKY59, or 100 nM for CFA0305. Sensor surface was regenerated using 25 mM NaOH. Kinetics parameters at both pH conditions were determined by fitting the sensorgrams with 1:1 binding model using Biacore T200 Evaluation software, version 2.0 (GE Healthcare). The other details are described in Supplementary Information.

### *In vivo* study using human FcRn transgenic mice

All animal care and experiments were performed in accordance with the guidelines for the care and use of laboratory animals at Chugai Pharmabody Research (CPR). The experimental protocols were approved by Institutional Animal Care and Use Committee of CPR. All procedures involving mice were carried out by licensed personnel. Solution containing human C5 (0.1 mg/kg) only or mixture of human C5 (0.1 mg/kg) and anti-human C5 antibody (20 mg/kg) was administered into human FcRn homozygous transgenic mice, line #32 (B6.16 mouse FcRn−/−, human FcRn transgenic line 32+/+ mouse, Jackson Laboratories) intravenously. The concentration of anti-human C5 antibody in mouse plasma was measured by electrochemiluminescence (ECL) assay. Anti-human IgG κ chain antibody (Antibody Solutions) was dispensed onto a MULTI-ARRAY 96-well plate (Meso Scale Discovery) and incubated overnight at 4 °C. Anti-human IgG antibody (Southernbiotech) with SULFO-Tag label was used as detection antibody. The signal was detected by Sector Imager 2400 (Meso Scale Discovery). The concentration of total human C5 in mouse plasma was measured by ECL assay. Anti-human C5 antibody (in-house) was dispensed onto a MULTI-ARRAY 96-well plate and incubated overnight at 4 °C. Anti-human C5 antibody (in-house) with SULFO-Tag label was used as detection antibody. The signal was detected by Sector Imager 2400 (Meso Scale Discovery). The concentration of total human C5 in mouse plasma was measured by ECL assay. Anti-human C5 antibody (in-house) was dispensed onto a MULTI-ARRAY 96-well plate and incubated overnight at 4 °C. Anti-human C5 antibody (in-house) with SULFO-Tag label was used as detection antibody. The signal was detected by Sector Imager 2400.

### Fluorescence imaging of cytoplasmic endosomes

The Fc region of the antibodies was converted to IgG1 with mutations (M252Y/N434Y/Y436V; EU numbering) to enhance FcRn binding at neutral pH to increase the sensitivity. Antibody-antigen complex was generated by incubating labeled-human C5 (Alexa Fluor® 555, Invitrogen) and antibodies at 37 °C CO_2_ incubator for 2 hours. For confocal microscopy experiment, MDCK cells (ATCC) stably expressing human FcRn-EGFP were grown on a Poly-D-Lysine coated 96-well glass-bottom plate (MatTek) one day before experiment. On the day of experiment, the cells were treated with medium containing pre-formed immune complex for 10–15 min. After treatment, cells were observed (Nikon A1+ confocal laser microscope, Nikon). For excitation, a laser excitation system was used consisting of 2 lasers: 488 nm and 561 nm to make an image of EGFP, and Alexa 555-labeled C5, respectively.

### *In vivo* study using cynomolgus monkeys

Animal care and experiments were performed in accordance with the guidelines for the care and use of laboratory animals at Maccine, Pte. Ltd. (Singapore) and Shin Nippon Biomedical Laboratories, Ltd. (SNBL, Japan). The experimental protocols were approved by Institutional Animal Care and Use Committee of Maccine and SNBL. All procedures involving monkeys were carried out by licensed personnel. Anti-human C5 antibodies were administered to cynomolgus monkeys. CFA0322 (40 mg/kg) was administered intravenously at day 0 and 14. SKY59 (20 mg/kg) was administered intravenously at day 0 or SKY59 (5 mg/kg) was administered intravenously at day 0 and subsequently SKY59 (2 mg/kg) was administered subcutaneously at day 7, 21, 35, 49, and 63. The concentration of anti-C5 antibodies in cynomolgus monkey plasma was measured by ELISA assay. Anti-human IgG κ chain antibody (Antibody Solutions) was dispensed onto a Nunc-Immuno 96-well plate MaxiSorp (Nalge Nunc International) and incubated overnight at 4 °C. Anti-human IgG antibody with HRP label (Southernbiotech) was used as detection antibody. The signal was detected by Multi-Skan™ GO. This ELISA format can detect total human IgG (free antibody + complex (antibody + C5)) using anti-human IgG antibodies as capture and detector. There was no interference by C5 in the ELISA, and both the IgG1 and IgG4 were confirmed to show comparable response in this assay system. The concentration of total monkey C5 in plasma was measured by ELISA assay for SKY59 and CFA0305. Anti-human C5 antibody (in-house) was dispensed onto a Nunc-Immuno 96-well plate MaxiSorp™ (Nalge Nunc International) and incubated overnight at 4 °C. Excess amount of SKY59 was added to samples so that all monkey C5 in samples bind to SKY59. Anti-human IgG antibody with HRP label (Southernbiotech) was used as detection antibody. The signal was detected by Multi-Skan™ GO (Thermo Scientific). The concentration of total monkey C5 in monkey plasma was measured by ECL assay for CFA0322. Anti-human C5 antibody (in-house) was dispensed onto a MULTI-ARRAY 96-well plate (Meso Scale Discovery) and incubated overnight at 4 °C. Anti-human C5 antibody (in-house) with SULFO-Tag label was used as detection antibody. The signal was detected by Sector Imager 2400 (Meso Scale Discovery). It was confirmed that there was no interference by SKY59, CHA0305, or CFA0322 in the assays.

### Hemolytic assay

The anti-C5 antibodies were tested for inhibition of complement activity in cynomolgus monkey plasma. cRBC (IC05-0810, Innovative research) were washed with gelatin/veronal-buffered saline containing 0.5 mM MgCl_2_ and 0.15 mM CaCl_2_ (GVB++) (IBB-300, Boston BioProducts), and thereafter sensitized with anti-chicken RBC antibody (103–4139, Rockland) for 15 min at 4 °C. The cells were then washed with GVB++ and suspended in the same buffer at 1 × 10^8^ cells/mL. In a round-bottom 96-well microtest plate, monkey plasma was incubated with the sensitized cRBC for 20 min at 37 °C. After centrifugation, supernatants were transferred to wells on a flat-bottom 96-well microtest plate for measurement of OD at 415 nm with a reference wavelength at 630 nm. The relative rates (R) of hemolytic activity at each post administration to that of pre administration was calculated from the following formula: R (%) = 100 × [(OD_Post administration_ − OD_Plasma and cRBC background_)]/[(OD_Pre administration_ − OD_Plasma and cRBC background_)].

### Data collection and structure determination

Diffraction data were collected on beamline BL32XU of SPring-8 at 95 K. Determining the cell parameters, indexing the diffraction spots, and processing the diffraction data obtained from the diffraction images were performed using the xia2^39^, which utilized Labelit^40^, XDS^41^, Aimless^42^, and Pointless^43^ in the CCP4 package^44^, and finally the diffraction intensity data up to 2.11 Å resolution were obtained. The structure was determined by molecular replacement with the program Phaser^45^. The search model of the Fab domain was derived from the published human IgG4 Fab crystal structure (PDB ID: 1BBJ)^46^, and that of the MG1 domain from the published human C5 crystal structure (PDB ID: 3CU7)^47^. A model was built with the Coot program^48^ and refined with the program Refmac5^49^. The structure has been deposited in the RCSB Protein Data Bank with PDB ID: 5B71. Molecular graphics figures were prepared using PyMOL (The PyMOL Molecular Graphics System, Version 1.7 Schrödinger, LLC.

The other details are described in Supplementary Information.

## Electronic supplementary material

Supplementary Information
