# Supplementary material for: Long lasting neutralization of C5 by SKY59, a novel recycling antibody, is a potential therapy for complement-mediated diseases
Source: Sci Rep. 2017 Apr 24;7:1080. doi: 10.1038/s41598-017-01087-7 (PMC5430875; doi:10.1038/s41598-017-01087-7)
Supplement: Supplementary file 1 — Supplementary Information [file 41598_2017_1087_MOESM1_ESM.docx]

Supplementary Information

**Long lasting neutralization of C5 by SKY59, a novel recycling antibody, is a potential therapy for complement-mediated diseases**

**Authors:**

Taku Fukuzawa^1^, Zenjiro Sampei^1^, Kenta Haraya^1^, Yoshinao Ruike^1^, Meiri Shida-Kawazoe^2^, Yuichiro Shimizu^1^, Siok Wan Gan^1^, Machiko Irie^3^, Yoshinori Tsuboi^2^, Hitoshi Tai^4^, Tetsushi Sakiyama^2^, Akihisa Sakamoto^2^, Shinya Ishii^2^, Atsuhiko Maeda^2^, Yuki Iwayanagi^3^, Norihito Shibahara^2^, Mitsuko Shibuya^2^, Genki Nakamura^2^, Takeru Nambu^2^, Akira Hayasaka^2^, Futa Mimoto^2^, Yuu Okura^1^, Yuji Hori^2^, Kiyoshi Habu^3^, Manabu Wada^3^, Takaaki Miura^3^, Tatsuhiko Tachibana^3^, Kiyofumi Honda^1^, Hiroyuki Tsunoda^3^, Takehisa Kitazawa^3^, Yoshiki Kawabe^2,3^, Tomoyuki Igawa^2^, Kunihiro Hattori^3^ , Junichi Nezu^1^*

**Affiliations:**

^1^Chugai Pharmabody Research Pte. Ltd., Singapore; ^2^Research Division, Chugai Pharmaceutical Co., Ltd., Gotemba, Shizuoka, Japan; ^3^Research Division, Chugai Pharmaceutical Co., Ltd., Kamakura, Kanagawa, Japan; ^4^Chugai Research Institute for Medical Science, Inc., Gotemba, Shizuoka, Japan.

*Correspondence and requests for materials should be addressed to J.N. (e-mail: NEZU.J@chugai-pharmabody.com).

**Supplementary Methods**

**Expression and purification of recombinant C5**

Recombinant human C5 (NCBI GenBank accession number: NP_001726.2) was used for experiments unless stated otherwise. Human C5 was expressed transiently using FreeStyle293-F cell line (Thermo Fisher). Conditioned medium expressing human C5 was applied to a Q-sepharose FF or Q-sepharose HP anion exchange column (GE healthcare), followed by elution with NaCl gradient. Fractions containing human C5 were pooled, then salt concentration and pH was adjusted to 80 mM NaCl and pH 6.4, respectively. The resulting sample was applied to a SP-sepharose HP cation exchange column (GE healthcare) and eluted with a NaCl gradient. Fractions containing human C5 were pooled and subjected to CHT ceramic Hydroxyapatite column (Bio-Rad Laboratories). Human C5 eluate was then applied to a Superdex 200 gel filtration column (GE healthcare). Fractions containing human C5 were pooled and stored at −150°C. Expression and purification of recombinant cynomolgus monkey C5 (NCBI GenBank accession number: XP_005580972) was done exactly the same way as the human counterpart. Human C5 variants were expressed by using FreeStyle293-F cell line, and the conditioned medium was used for the study.

**Expression and purification of recombinant antibodies**

Recombinant antibodies and h5G1.1 G2/G4 (eculizumab) were expressed transiently using FreeStyle293-F cell line (Thermo Fisher). Purification from the conditioned medium expressing antibodies was done with conventional method using protein A. Gel filtration was further conducted if necessary.

**Antibody generation**

Twelve- to sixteen-week-old NZW rabbits were immunized intradermally with human C5 (Merck Millipore) (50-100 µg/dose/rabbit). This dose was repeated 4-5 times over a 2 month period. One week after the final immunization, spleen and blood samples were collected from the immunized rabbits. Antigen-specific B-cells were stained with labeled antigen, sorted using a cell sorter (FACS aria III, BD), and co-cultured for 7-12 days in 96-well plates at one cell/well density together with 25,000 cells/well of EL4 cells (European Collection of Cell Cultures) in diluted (20-fold), activated rabbit T-cell conditioned medium. EL4 cells were pre-treated with mitomycin C (M4287, Sigma) for 2 hours and washed 3 times before culture. After cultivation, the B-cell culture supernatant was collected for further analysis and the cell pellets were cryopreserved. Activated rabbit T-cell conditioned medium was prepared by culturing rabbit thymocytes in RPMI-1640 containing Phytohemagglutinin-M (1 1082132-001, Roche), phorbol 12-myristate 13-acetate (P1585, Sigma) and 2% FBS.

**Antibody screening**

ELISA assay was used to test the specificity of antibodies in B-cell culture supernatant. Streptavidin (Z02043, GenScript) was coated onto 384-well MaxiSorp (164688, Nunc) plates at 50 nM concentration in PBS for 1 hour at room temperature. Plates were then blocked with diluted (5-fold) Blocking One (03953-95, Nacalai Tesque). Human C5, pre-labeled with NHS-PEG4-Biotin (21329, PIERCE), was added to the ELISA plates after the blocking step for a 1 hour incubation. Then the plates were washed and the B-cell culture supernatants were added next for a 1 hour incubation. Then the plates were washed and binding was detected by goat anti-rabbit IgG-Horseradish peroxidase (A120-111P, BETHYL) followed by the addition of ABTS (50-66-06, KPL). ELISA assay was used to evaluate pH-dependent binding of antibodies against C5. After incubation with biotinylated human C5, plates were washed and incubated with either pH 7.4 MES buffer (20 mM MES, 150 mM NaCl and 1.2 mM CaCl_2_) or pH 5.8 MES buffer (20 mM MES, 150 mM NaCl and 1 mM EDTA) for 1 hour at room temperature. After incubation, binding of biotinylated C5 was detected by Streptavidin-Horseradish peroxidase conjugate (21132, Thermo Scientific) followed by the addition of ABTS.

**Biacore binding assays**

Binding kinetic of anti-C5 antibodies against human C5 or cynomolgus monkey C5 were assessed at pH 7.4 and pH 5.8, at 37°C using Biacore T200 instrument (GE Healthcare). ProA/G (Pierce) was immobilized onto C1 sensorchip using amine coupling kit (GE Healthcare) according to the recommended settings by GE Healthcare. Antibodies and analytes were diluted into the respective running buffers, ACES pH 7.4 and pH 5.8 (20 mM ACES, 150 mM NaCl, 1.2 mM CaCl_2_, 0.05% Tween 20, 0.005% NaN_3_). Each antibody was captured onto the sensor surface by ProA/G. Recombinant C5 was prepared by two-fold serial dilution started from 12.5 nM for SKY59, or 100 nM for CFA0305. The surface was regenerated using 25 mM NaOH. Kinetic parameters at both pH conditions were determined by fitting the sensorgrams with 1:1 binding model using Biacore T200 Evaluation software, version 2.0 (GE Healthcare). Biacore binding analysis for human C5 mutation sample at residues E48, G51, and K109 were performed at pH 7.4 at 37°C using Biacore T200 instrument (GE Healthcare). ProA/G was immobilized onto CM4 sensorchip using amine coupling kit (GE Healthcare) according to the recommended settings by GE Healthcare. Antibodies were captured on different flow cells by proA/G. Culture supernatants containing the C5 variants were prepared at 40 µg/mL. For Biacore binding analysis, the sample was diluted (10-fold) with Biacore running buffer (Aces pH 7.4, 1 mg/mL Casein, 1 mg/mL carboxymethyl dextran) to a final sample concentration of 4 µg/mL. At the end of each analysis cycle, the sensor surface was regenerated with 25 mM NaOH. The results were analyzed in Bia Evaluation software, version 2.0 (GE Healthcare). The pH-dependent interaction assessment for C5 His mutation samples at residues H70Y, H72Y, H110Y, and H70Y + H110Y was determined by a modified Biacore assay. Briefly, an additional dissociation phase at pH 5.8 was integrated into the Biacore assay immediately after dissociation phase at pH 7.4. This is to assess the pH-dependent dissociation between antibody and antigen from the complexes formed at pH 7.4. The dissociation rate at pH 5.8 buffer was determined by processing and fitting data using Scrubber 2.0 (BioLogic Software) curve fitting software. Binding affinity of SKY59 against FcRn was assessed at pH 6.0 at 25°C using Biacore T200 instrument (GE Healthcare). Purified mouse anti-human Ig κ light chain (anti-human Igκ) (BD Biosciences) was immobilized onto CM5 sensorchip using amine coupling kit (GE Healthcare) according to the recommended settings by GE Healthcare. SKY59 and analytes were diluted into the running buffers, PB pH 6.0 (50 mM sodium phosphate, 150 mM NaCl, 0.05% Surfactant P20). SKY59 was captured onto the sensor surface by anti-human Igκ. The sensor surface was regenerated with glycine 2.0 (GE healthcare). To calculate the *K*_D_ values, Steady State Affinity fitting was carried out using Bia Evaluation software, version 2.0 (GE Healthcare).

**Preparation of C5 MG1 domain and SKY59 Fab fragment complex**

The MG1 domain (amino acid residues 20-124 of human C5) fused to a GST-tag via thrombin cleavable linker (GST-MG1) was expressed in the *E. coli* strain BL21 DE3 pLysS (Promega) using a pGEX-4T-1 vector (GE healthcare). Protein expression was induced with 0.1 mM Isopropyl β-D-1-thiogalactopyranoside for 5 hours at 25°C. The bacterial cell pellet was lysed with Bugbuster (Merck) supplemented with Lysonase (Merck) and cOmplete^TM^ protease inhibitor cocktail (Roche), followed by the purification of GST-MG1 from the soluble fraction using a GSTrap column (GE healthcare) according to the manufacturer's instruction. The GST tag was cleaved with thrombin (Sigma), and the resulting MG1 domain was further purified with a Superdex 75 gel filtration column (GE healthcare). Fab fragment of SKY59 was prepared by the conventional method using limited digestion with papain (Roche Diagnostics), followed by loading onto a protein A column (MabSlect SuRe, GE Healthcare) to remove Fc fragment, a cation exchange column (HiTrap SP HP, GE Healthcare), and a gel filtration column (Superdex 200 16/60, GE Healthcare). Purified recombinant human C5 MG1 domain was mixed with a purified SKY59 Fab fragment in a 3:2 molar ratio. The complex was purified with a gel filtration column (Superdex 200 10/300 increase, GE Healthcare) equilibrated with 25 mM HEPES pH 7.5, 100 mM NaCl in order to eliminate the excess C5 MG1.

**Crystallization**

The purified complexes were concentrated to about 10 mg/mL with 5 kDa cut-off polyethersulfone membrane (Vivaspin 20 MWCO 5,000, GE Healthcare), and crystallization was carried out by the sitting drop vapor diffusion method at 4°C in combination with the seeding method. The reservoir solution consisted of 0.2 M magnesium formate dehydrate and 15.0% w/v polyethylene glycol 3350. This succeeded in yielding plate-like crystals in a few days. The crystal was soaked in a solution of 0.2 M magnesium formate dehydrate, 25.0% w/v polyethylene glycol 3350, and 20% glycerol for data collection.

**Supplementary Figures and Table**

Supplementary Fig. 1.

a b

c

**Supplemental Figure 1. Inhibition of MAC formation by eculizumab and SKY59**. (**a**-**c**) Inhibitory activity of eculizumab and SKY59 on MAC formation in the CP (**a**), AP (**b**), and LP (**c**) was evaluated by ELISA with normal human serum. The serum concentrations are 0.5%, 5.5%, and 1% for CP, AP, and LP, respectively. SKY59 and eculizumab inhibited MAC formation in all complement pathways. Data are presented as means ± S.D.

Supplementary Table 1.

| **Data collection** |  |
| --- | --- |
| Space group | *P*1 |
| Unit Cell |  |
| *a,b,c* (Å) | 39.79, 55.10, 127.76 |
| α,β,γ (°) | 89.18, 86.24, 78.20 |
| Resolution (Å) | 42.49–2.11 |
| Total reflections | 112,102 |
| Unique reflections | 56,154 |
| Completeness (highest resolution shell) (%) | 92.1 (95.8) |
| *R*_merge_ ^a^ (highest resolution shell) (%) | 7.2 (31.7) |
| **Refinement** |  |
| Resolution (Å) | 25.00–2.11 |
| Reflections | 53,398 |
| *R* factor ^b^ (*R*_free_ ^c^) (%) | 24.59 (28.53) |
| rms deviation from ideal |  |
| Bond lengths (Å) | 0.0069 |
| Bond angles (°) | 1.0620 |

a; *R*_merge_ = ∑*hkl*∑*j*|*Ij* (*hkl*) − 〈*I* (*hkl*)〉|/∑*hkl*∑*j*|*Ij* (*hkl*)|, where *Ij* (*hkl*) and 〈*I* (*hkl*)〉 are the intensity of measurement *j* and the mean intensity for the reflection with indices *hkl*, respectively.

b; *R* factor = ∑*hkl*|*F*_obs_ (*hkl*)| − |*F*_calc_ (*hkl*)|/∑*hkl*|*F*_obs_ (*hkl*)|, where *F*_obs_ and *F*_calc_ are the observed and calculated structure factor amplitudes, respectively.

c; *R*_free_ is calculated with 5% of the reflection randomly set aside.

Supplementary Table 1. X-ray data collection and refinement statistics.

Supplementary Fig. 2.

a


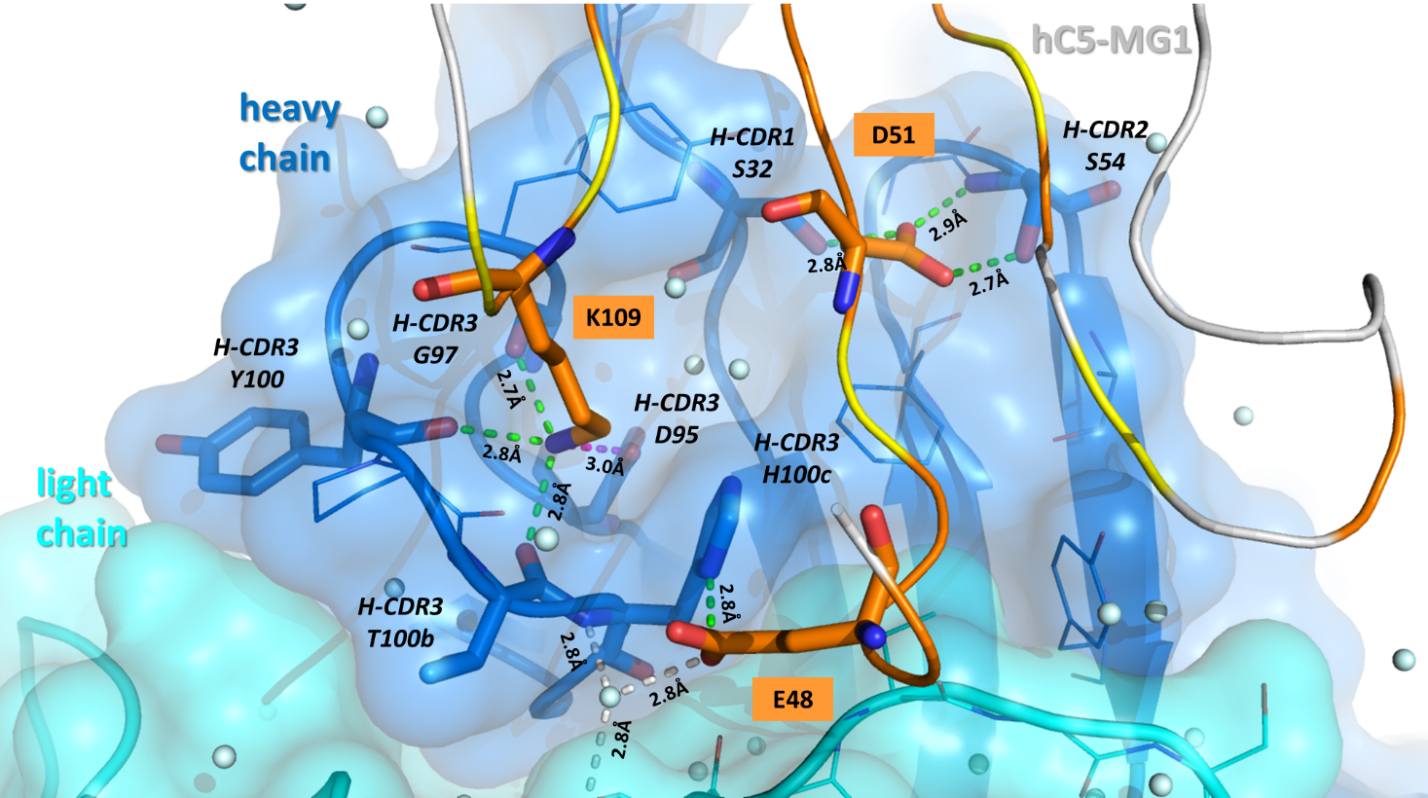


b


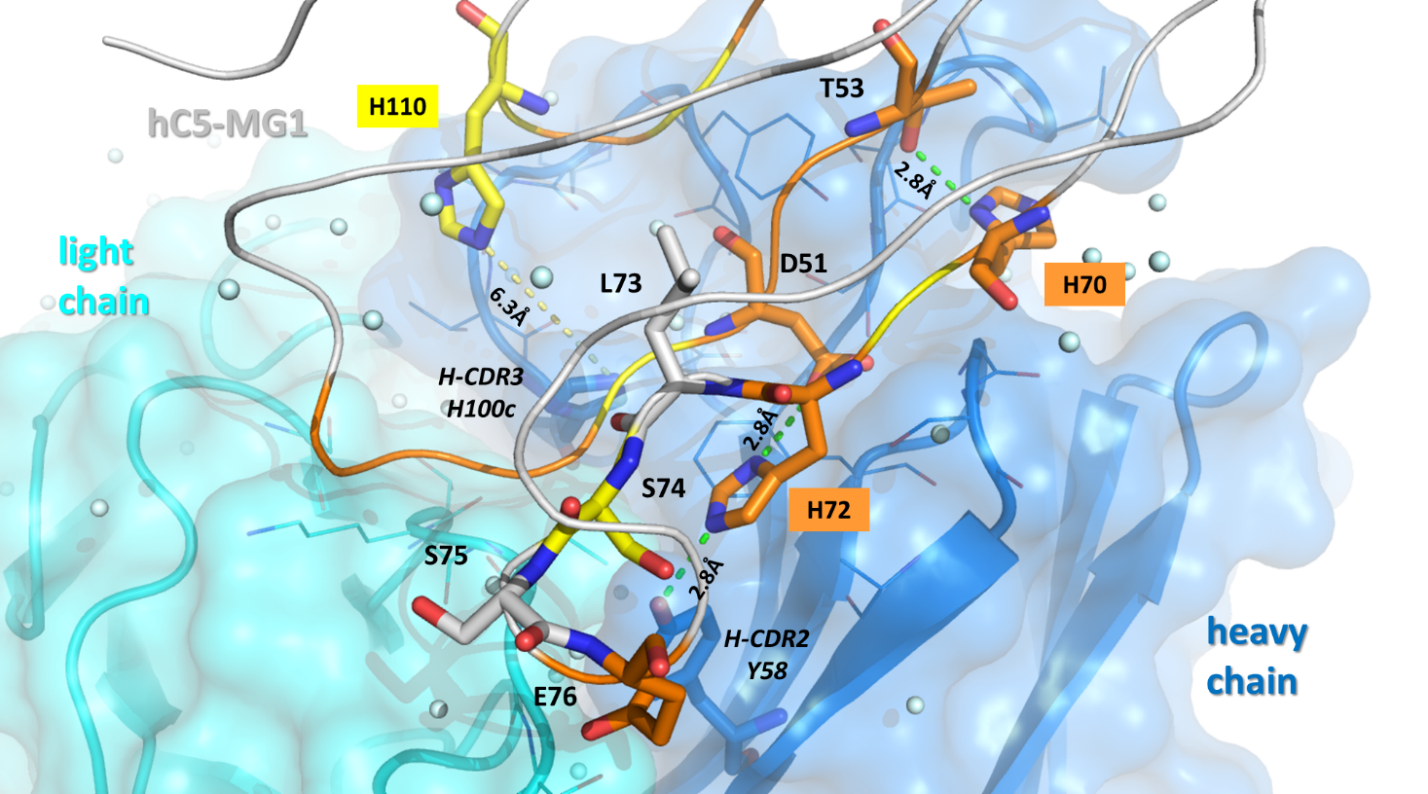


**Supplementary Figure 2. A close-up view of the interactions of MG1 with SKY59 Fab.** SKY59 Fab and MG1 residues are colored the same as in Fig. 5b. (**a**) Interactions between SKY59 Fab and the side chains of three amino acids: E48, D51, and K109 (labeled in orange). Direct hydrogen bonds with SKY59 and water-mediated hydrogen bonds are indicated by green and white dotted lines, respectively. A salt bridge between K109 and H_CDR3 D95 is indicated by the magenta dotted line. (**b**) Interactions between each histidine residue (H70 and H72 labeled in orange, and H110 labeled in yellow) and its environment are illustrated. Hydrogen bonds are indicated by green dotted lines. The distance between H110 and H-CDR3_H100c is shown by a yellow dotted line.

Supplementary Fig. 3.

a

SKY59


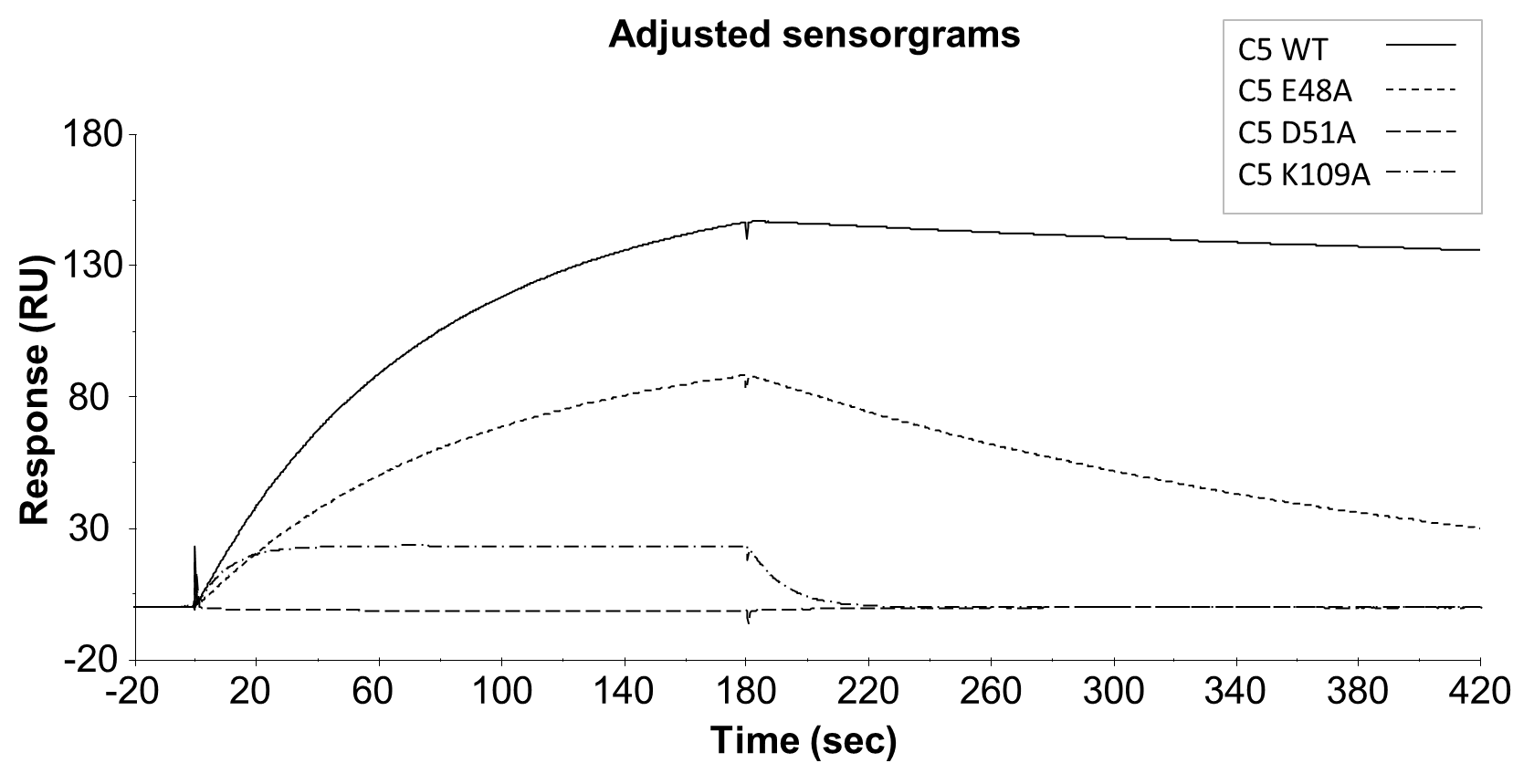


Eculizumab


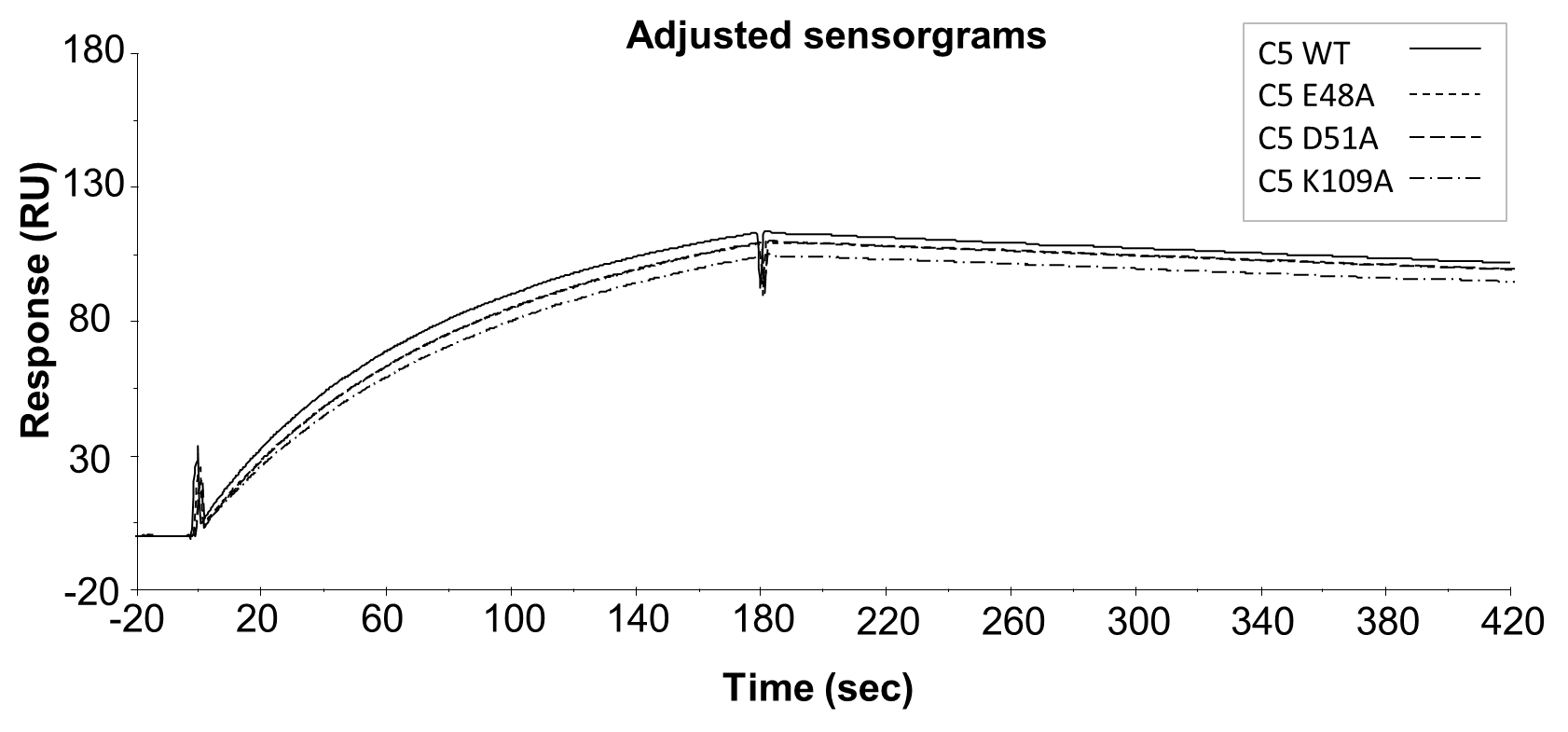


b


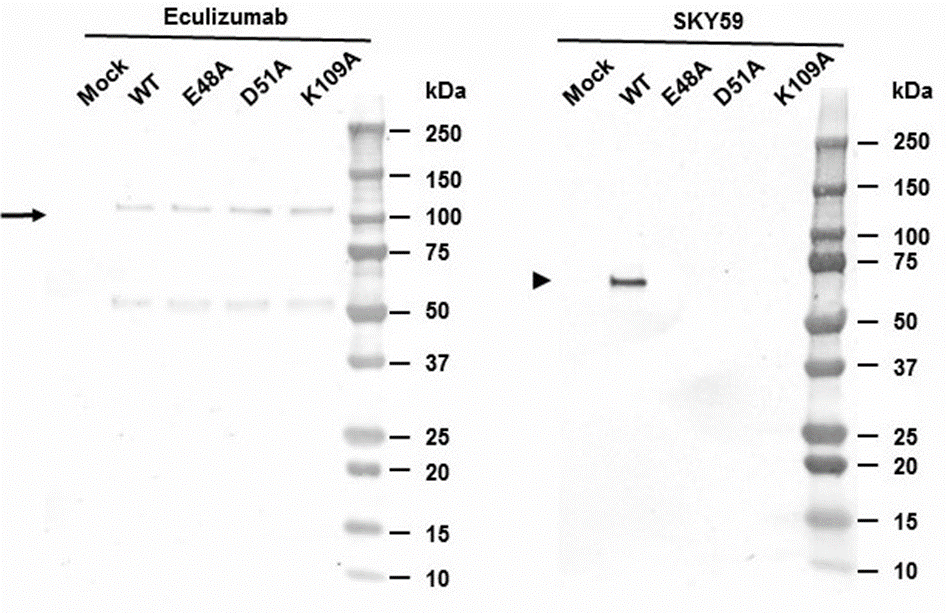


**Supplementary Figure 3. Interaction of eculizumab and SKY59 with C5 variants.** (**a**) Biacore sensorgrams were obtained by injection of C5 WT (thick solid curve), C5 E48A (short-dashed curve), C5 D51A (long-dashed curve), and C5 K109A (dashed dotted curve), respectively, over sensor surface captured with SKY59 or eculizumab. (**b**) Western blot analysis was performed with C5 WT and the point mutants. In the left panel, eculizumab was used for the reaction. The position of the α-chain of C5 (approx.113 kDa) is marked with an arrow. In the right panel, SKY59 was used for the reaction. The position of the β-chain of C5 (approx. 74 kDa) is marked with an arrowhead.
